# Application of Graphene-Based Materials

**DOI:** 10.3390/nano13202748

**Published:** 2023-10-12

**Authors:** Marcelo Antunes

**Affiliations:** Department of Materials Science and Engineering, Poly2 Group, Technical University of Catalonia (UPC BarcelonaTech), ESEIAAT, C/Colom 11, 08222 Terrassa, Spain; marcelo.antunes@upc.edu; Tel.: +34-93-7398056

This Topic on the “Application of Graphene-Based Materials”, which consists of a total of twenty-six articles, including two review articles, written by research groups of experts in the field, considers the most recent research and trends on the synthesis and characterization of graphene-based materials, including nanohybrids, intended for a vast array of high-demanding technological applications, namely batteries/fuel cells, aerogels, laser technology, sensors, electronic/magnetic devices, catalysts, etc.

One of the reviews included in the present Topic considers the most recent research of graphene-based materials, with the authors even calling it “graphene technology”, to be used in lasers [1]. Namely, the authors claimed the interest in recent years for the development of laser-induced graphene (LIG) technology as an easier way of guaranteeing the formation of patterned, controlled graphene-based structures, especially when compared to traditional graphene preparation methods, opening a broader range of possible high-technological applications such as supercapacitors, sensors, or filters, just to name a few examples. This extensive review summarizes a great number of preparation methods for graphene-based materials, focusing on the effects of the laser processing characteristics, the possibility of establishing strategies for large-scale LIG production, and their viable use for energy storage and signal sensing purposes. Related to this review article, Fiodorov and co-workers [2] have reported a laser-assisted method for the selective deposition of copper on polymer-based surfaces via electroplating, based on the formation of a graphene-like layer on the surface of the polymer after the laser treatment and before the electroplating deposition of the copper layer, which, according to the authors, could ultimately enable the “copper track printing” on dielectric devices having a complex shape and, hence, the manufacturing of molded interconnect devices (MIDs).

The unique characteristics of graphene-based materials make them ideal candidates to be considered for energy storage purposes and in the high-trending market of batteries/fuel cells. Guo et al. [3] considered the use of single, bi, tri, and up till tetra-layer graphene sheets in the development of a high-energy density anode, crucial in the practical application of boron ion portable batteries. In this work, the authors demonstrated that it is possible to tailor and control the performance of graphene-based anodes in boron ion batteries by increasing the number of graphene layers, reaching cell voltage values that enable the practical use of these batteries, even as replacers of the more common Li ion ones. Yaldagard and co-workers [4] used coated graphene nanoplatelets as supporters for platinum electrocatalysts in direct ethanol fuel cells, and demonstrated the high activity of the Pt-coated graphene material for CO electrooxidation, its high catalytic activity for the EOR, and its lower charge transfer resistance in the presence of ethanol (i.e., enhanced ethanol oxidation activity), all essential aspects in this kind of fuel cell.

Due to its pressure-sensitive properties, graphene (and its derivatives) has found interest in the development of sensors, and, due to its highly tunable electrical conductive properties, more specifically, piezoresistive sensors. Florencio and co-workers [5] have prepared and evaluated prototypes of graphene-based piezoresistive sensors, namely based on graphene papers prepared from different routes for measuring pressures up to 2 kPa. The authors found out that among all possibilities, sensors prepared from graphene oxide showed the highest sensitivity to pressure. In a similar way, Abedi et al. [6] investigated the piezoresistivity of a geocomposite, analyzing how the electrical resistivity may affect the piezoresistivity performance using different concentrations of carbon-based nanofillers, including graphene nanoplatelets. They demonstrated that, generally, increasing the aspect ratio of the nanofillers causes a reduction in the critical concentration for electrical conduction (percolation threshold) of the geocomposite, enhancing its efficiency as piezoresistive sensor and widening its applicability to field-applicable, self-sensing components. In a much more specific way, but with a similar objective of using the unique characteristics of graphene to develop sensors, Liu and co-workers [7] developed and characterized a waveguide comprising double-layer graphene, intended to excite and manipulate mid-infrared electromagnetic waves, showing their viability to be used in refractive index sensors and in future photonic integrated circuits.

One of the most interesting uses of graphene-based materials in the field of materials science is in the development of ultralight aerogels for different applications, from enhanced wave absorption to liquid adsorption. In this sense, Mei et al. [8] considered the development of graphene-based aerogels with enhanced microwave absorption, a trending topic due to the growing electromagnetic pollution coming from the simultaneous use of electronic devices. Interestingly, they were able to create a gradient-like open-cell graphene aerogel with an extremely high microwave absorption value of 62.6 dB only using 0.53 wt% graphene, which they explained based on a multiple reflection effect due to the open-cell structure and well-matched impedance due to its gradient structure. Taking advantage of the particular structure of aerogels and, hence, possible highly efficient adsorption capabilities, Jin and co-workers [9] developed bionanocomposite aerogels containing graphene oxide for the removal of dyes, specifically methylene blue, from wastewater. Particularly, they were able to develop aerogels that spontaneously and efficiently adsorbed methylene blue. In their extensive review, Joya-Cárdenas et al. [10] assessed the capacities of graphene-based materials, namely based on graphene oxide, for the adsorption of water contaminants, such as arsenite, arsenate, fluoride, and hexavalent chromium. This study displayed the advances that have been performed in this field, as well as the development of novel graphene oxide-based materials, focusing on their synthesis methods and relation with the adsorption capacity, and their current and future perspectives. It has been demonstrated that the adsorption and selectivity capabilities of graphene-based materials can be highly tunable via prior surface modification of graphene layers; this is deemed desirable, owing to the intrinsic flat-like high surface area of graphene. In this sense, Rodríguez-Pastor’s research group [11] developed an effective method for preparing graphene oxide with extremely high selectivity in carboxyl groups, highly desirable for applications requiring strong interactions between bioactive molecules and a given substrate, as is the case for the biomedical field. Taking advantage of this situation, Barjola et al. [12] prepared carboxylated graphene oxide–silver nanoparticles and studied their antibacterial and biofilm formation inhibitions, showing that the presence of carboxyl groups inhibited the aggregation of silver nanoparticles and led to lower inhibitory concentrations and minimum bactericidal concentrations for all analyzed strains when compared to non-carboxylated graphene oxide–silver nanoparticles, as well as to higher reductions in biofilm mass and cell viability.

Owing to their highly interesting transport properties, namely high and tunable electrical conductivity and magnetic characteristics, graphene-based materials have generated a great deal of interest in applications where electron charge transport may be of crucial importance, as in electronics. For instance, the group of Roshchupkin [13] investigated the use of acoustic waves to stimulate charge transport in graphene films, showing that it is possible to control both the value and the direction of the current in the graphene film by controlling the excitation frequency and amplitude of the surface acoustic wave. The authors claimed that this new method of charge collection and transfer will considerably enhance the still low efficiency of solar cells in the future. Archana and co-workers [14] took advantage of the semi-metallic properties of graphene by supporting graphone, resulting from the semi-hydrogenation of graphene sheets, on a nickel surface, also intercalating Al or Na atoms. In this way, they were able to tune the interface properties between nickel and graphone surfaces, with the resulting assemblies finding their possible use as spintronic or semiconducting materials, for instance, for developing smart devices. Suo et al. [15] developed a cost-effective simulation method to assess the properties of graphene-intercalated compounds, such as the ones mentioned before.

In addition to their highly desirable characteristics for charge transport, graphene-based materials also display theoretically high mechanical characteristics, such as high strength, while keeping a significant flexibility. As a consequence, especially in the field of materials science, a great number of research groups have focused their interest in using graphene or its derivatives as nanoreinforcements, alone or in combination with other (nano)reinforcements, in different types of matrices, especially polymer-based. An example of this use for graphene can be seen in the work of Lim and co-workers [16], which considered the use of graphene nanoplatelets to mechanically reinforce polyamide 610. These researchers have focused on the influence of the amount of added graphene and the type of thermal treatment applied to graphene prior to its addition to PA610 in the tensile strength of the resulting nanocomposites, showing that a maximum tensile strength could be reached until graphene nanoplatelets started to form aggregates (concentrations > 1.5 wt% graphene). Polyimide nanocomposite films containing functionalized graphene and organoclay hybrids were prepared by Lee et al. [17] at loadings between 0.25 and 1.0 wt%. These authors were able to keep the characteristic optical transparency of polyimide while enhancing its thermo-mechanical performance, enabling the use of these films in electronic and optical applications, two of the most trending fields in materials science. One possible application of graphene-based materials that is directly related to their high mechanical performance, namely surface mechanical properties such as wear strength, is orthodontics. Wang et al. [18] studied the effects of adding graphene to carbon films on the fretting friction and wear behavior of the resulting films for common orthodontic stainless steel archwire brackets. These authors demonstrated that the use of graphene and the combination of graphene-modified films with the mentioned brackets led to components with exceptional friction and wear behavior, suggesting a great potential for clinical orthodontics. Hussein [19] further extended this concept by assessing the possible use of graphene-based polymers as esthetic clasp materials. Although the developed graphene-based materials displayed a worse mechanical performance as a clasp material when compared to PEEK, the author of this manuscript concluded that an optimization study is still required to check the validity of the use of graphene in said application. Taking advantage of the intrinsic lubrication properties of graphene and, hence, promising characteristics to reduce the friction and wear in nanocomposites, Yallew and co-workers [20] reported how the fabrication process may affect the frictional and wear strength of graphene-coated polymers. These authors showed how graphene coats effectively enhance both frictional and wear strength of polymer substrates, with the final enhancement depending on graphene’s transfer method, comparatively dry-transferred graphene resulting better than wet-transferred graphene, and the quality of the adhesion between the graphene coating and the polymer substrate.

A high interest has been dedicated to the possible use of graphene-based materials as part of a (nano)catalyst system. An example of this use is the work of Zhao and colleagues [21], which has considered the synthesis of a novel ternary nanocomposite prepared using the combination of AgZnS, TiO_2_, and reduced graphene oxide. The nanocomposite showed an extremely high level of photocatalytic activity through hydrogen production by water splitting, far exceeding that of undoped TiO_2_ and reduced graphene oxide, hence showing promising application as hydrogen evolution catalyst.

Alongside all the abovementioned possibilities of graphene-based materials, there are others that, although less typical, are also being considered by researchers, presenting another example of the versatility of graphene. Teshigawara et al. [22] proposed the use of nanosized graphene aggregates to enhance neutron intensity below cold neutrons, overcoming some of the current problems of using nanodiamonds in practical applications, particularly processing/molding of the components, and demonstrating its viability, most likely due to coherent scattering, to be used in below cold neutron applications. Another attractive characteristic of graphene is that its Fermi level may be tailored via doping. Fan and co-workers [23] focused on the development of a theoretical model to describe the characteristics of the thermal emission of graphene versus gate voltages to be used as thermal emitters. Alongside corroborating the viability of the theoretical model with experimental results, said model provides a simple method to predict the behavior of in-gate controlled long channel graphene devices.

Owing to its particular flat-like surface, together with its high specific area, graphene materials have been considered as interlayers in the development of several devices. An example of this can be seen in the work of Badokas and co-workers [24], in which they considered the growth of non-porous, high-quality GaN epilayers on GaN/sapphire templates using different graphene interlayer arrangements, from single layer to triple stack of monolayer graphene, for high-demanding applications such as ultraviolet photodetectors. Somewhat related, as it deals with the development of vertically aligned graphene-based substrates (VGSs) with copper coverage to be used in combination with GaN in field effect transistors, Aizawa et al. [25] presented their particular work on enhancing the robustness against thermal gradients of vertically aligned graphene by mechanically anchoring copper rib structures into the micro-grooves of VGSs. The proposed design homogenizes the thermal spreading behavior in heated GaN chips, improving their mechanical integrity and robustness during severe thermal transients.

Less seen, but also considered, are the possible applications of graphene related to food. In this Topic, the study of Peng et al. [26] dealt with the possible use of graphene oxide, together with pyraclostrobin, as a nanocarrier for the development of nanopesticides for agricultural purposes, particularly for avoiding plant fungal pathogens, demonstrating its efficiency and expanding the applicability of graphene-based materials to sustainable agriculture.
